# P-1547. Risk factors related to immune response, microbiome, and metabolomics in the progression of SARS-CoV-2 infection in cancer patients with COVID-19

**DOI:** 10.1093/ofid/ofaf695.1727

**Published:** 2026-01-11

**Authors:** Jayr Schmidt-Filho, Flávio Oshiro, Hans Obando, Alexandre Defelicibus, Gustavo Silva, Diana Nunes, Emmaneul Dias-Neto, Ivan França-Silva, Dirce Carraro, Israel Tojal, Valéria Simionato, Kenneth Gollob, Marjorie V Batista

**Affiliations:** A.C. Camargo Cancer Center, São Paulo, Sao Paulo, Brazil; Hospital Israelita Albert Einstein, São Paulo, Sao Paulo, Brazil; Universidade de Campinas, Campinas, Sao Paulo, Brazil; A.C. Camargo Cancer Center, São Paulo, Sao Paulo, Brazil; Hospital Israelita Albert Einstein, São Paulo, Sao Paulo, Brazil; A.C. Camargo Cancer Center, São Paulo, Sao Paulo, Brazil; A.C. Camargo Cancer Center, São Paulo, Sao Paulo, Brazil; A.C. Camargo Cancer Center, São Paulo, Sao Paulo, Brazil; A.C. Camargo Cancer Center, São Paulo, Sao Paulo, Brazil; A.C. Camargo Cancer Center, São Paulo, Sao Paulo, Brazil; Universidade de Campinas, Campinas, Sao Paulo, Brazil; Hospital Israelita Albert Einstein, São Paulo, Sao Paulo, Brazil; Department of Infectious Diseases, AC Camargo Cancer Center, São Paulo, SP, Brazil., São Paulo, Sao Paulo, Brazil

## Abstract

**Background:**

Risk factors for severe COVID-19 include age, comorbidities, and viral virulence. Despite vaccination reducing severe cases, immunocompromised patients remain at high risk. This study aimed to identify risk factors for SARS-CoV-2 progression based on immune response, microbiome, and metabolomic profiles in patients undergoing hematopoietic cell transplantation (HCT) or diagnosed with hematologic malignancies (HM) or solid tumors (ST).

**Figure 1.**
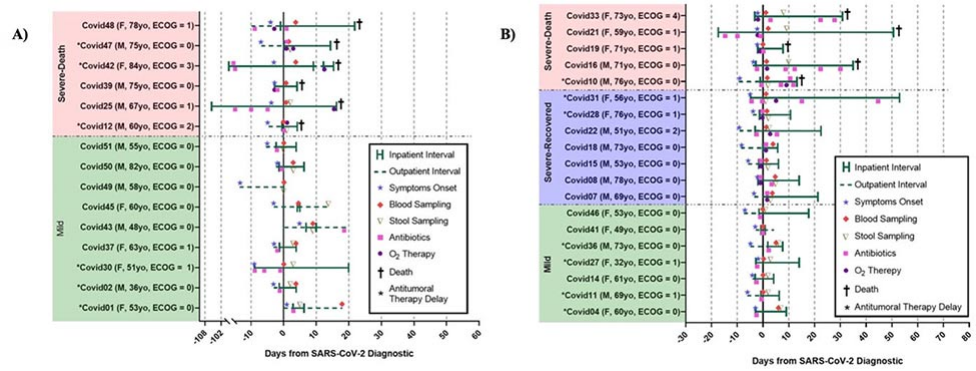
Clinical characteristics of the patient cohort with (A) Hematopoietic Cell Transplantation (HCT) or Hematologic Malignancies (HM), and (B) Solid Tumors (ST).

**Figure 2.**
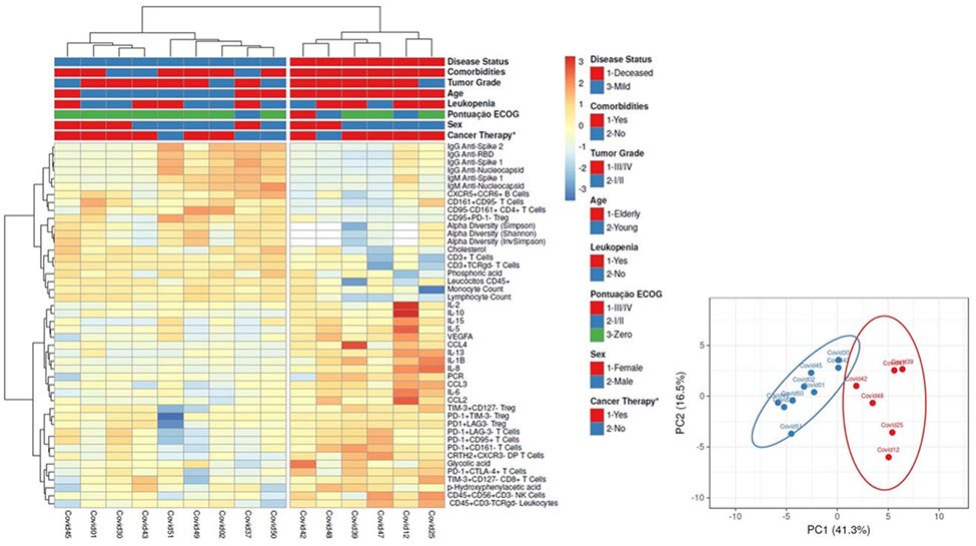
Integrated analysis of immune response, metabolomic, and metagenomic data in patients with Hematopoietic Cell Transplantation (HCT) or Hematologic Malignancies (HM).

**Methods:**

In this prospective cohort (April–August 2020), immune profiling included: (1) high-dimensional flow cytometry (T/B/myeloid cell activation, exhaustion, regulation); (2) cytokine/chemokine quantification; (3) ELISA for IgG/IgM against viral antigens. Microbiota composition was assessed via 16S rRNA sequencing from stool samples; plasma metabolomics was performed via GC-MS.

**Figure 3.**
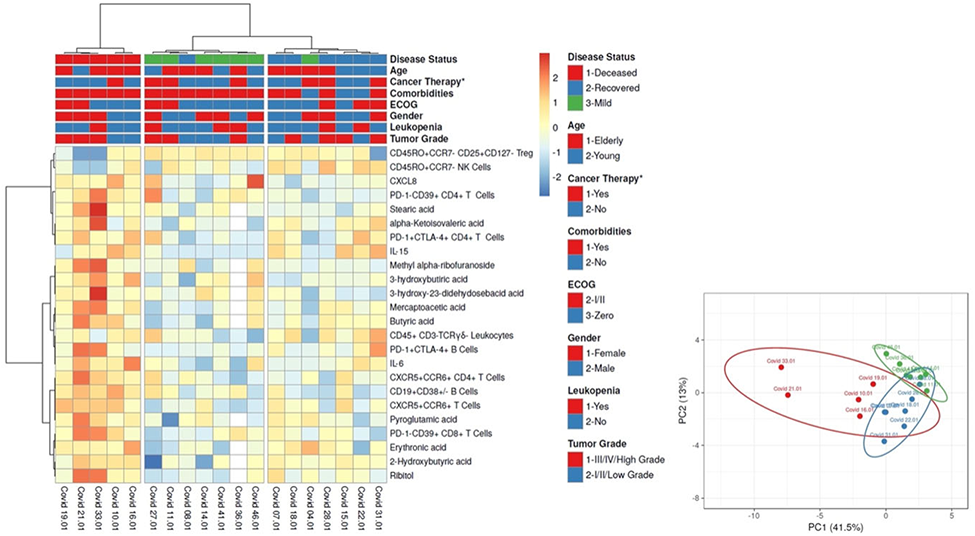
Integrated analysis of immune response and metabolomic data in patients with Solid Tumors(ST).

**Results:**

HCT or HM patients who died showed severe leukopenia, low anti-SARS-CoV-2 antibody levels, and increased innate immune activation, with IL-2, IL-13, TNF, IFN-γ, and FGF2 as cytokine hubs. HCT or HM patients who recovered had coordinated antibody networks involving IL-4, IL-5, IL-12A, IL-15, and IL-17A. ST patients who died showed high IL-6 and CXCL8 without leukopenia and lacked coordinated antibody responses. Those who recovered displayed IL-10-correlated regulatory cytokine networks. Alpha diversity was lower in HM or HCT patients with severe COVID-19 but not in ST. Distinct metabolic profiles were observed by GC-MS across mild, moderate, and severe cases.

**Conclusion:**

HCT or HM, and ST patients showed distinct immune, microbial, and metabolic signatures linked to COVID-19 outcomes. Integrative analysis may help identify high-risk cancer patients and guide future targeted management strategies.

**Disclosures:**

All Authors: No reported disclosures

